# A Case of Microangiopathic Antiphospholipid-Associated Syndromes during Pregnancy: Review of the Literature

**DOI:** 10.1155/2012/827543

**Published:** 2012-06-28

**Authors:** Nobuhiro Suzumori, Shintaro Obayashi, Kyoko Kumagai, Shinobu Goto, Atsuhiro Yoshida, Mayumi Sugiura-Ogasawara

**Affiliations:** ^1^Department of Obstetrics and Gynecology, Nagoya City University, Graduate School of Medicine, Nagoya 467-8601, Japan; ^2^Division of Clinical and Molecular Genetics, Nagoya City University, Graduate School of Medicine, Nagoya 467-8601, Japan; ^3^Department of Cardio-Renal Medicine and Hypertension, Nagoya City University, Graduate School of Medicine, Nagoya 467-8601, Japan

## Abstract

Microangiopathic antiphospholipid-associated syndromes (MAPSs) are reported as encompassing several conditions mainly affecting the microvasculature of selected organs: the liver in HELLP syndrome (hemolysis, elevated liver enzymes, and low platelet); kidney, brain, and skin in TTP (thrombotic thrombocytopenic purpura). It is predominant in patients with catastrophic antiphospholipid syndrome (APS). A recent report suggests that APS is not only a thrombotic disease but also associated with microangiopathic features, and it can explain the greater prevalence of HELLP syndrome in these patients. We here report a case of MAPS during pregnancy associated with systemic lupus erythematosus (SLE) in early second trimester.

## 1. Introduction

Obstetrical complications in revised classification criteria for the antiphospholipid syndrome (APS) include three or more unexplained spontaneous abortions before 10 weeks of gestation, intrauterine fetal death, and one or more premature births of a morphologically normal neonate before 34 weeks of gestation with continuous antiphospholipid (aPL) antibodies present in plasma [1]. APS is clearly related to maternal morbidity, and it is known to be a major cause of fetal loss due to a thrombotic tendency leading to placental infarction during pregnancy [2].

A recent report suggests that APS is not only a thrombotic disease but also associated with microangiopathic features, and it can explain the greater prevalence of HELLP (hemolysis, elevated liver enzymes, and low platelet count) syndrome in these patients [3]. Hepatic infarction, retinal vascular occlusions, and deep venous thrombosis (DVT) have now been reported in patients with HELLP syndrome [4]. Microangiopathic antiphospholipid-associated syndromes (MAPSs) are proposed as encompassing several conditions mainly affecting the microvasculature of selected organs: the liver in HELLP syndrome; kidney, brain, and skin in TTP (thrombotic thrombocytopenic purpura) and disseminated intravascular coagulation (DIC) [5]. 

Among HELLP cases diagnosed antepartum, 90% are in the third trimester and the syndrome rarely occurs before 27 weeks of gestation [6]. However, it is wellknown that several patients with APS suffer HELLP syndrome at very early onset despite low-dose aspirin and heparin combined therapy [7]. We here report a case of MAPS during pregnancy associated with systemic lupus erythematosus (SLE) in early second trimester. 

## 2. Case Report

A 39-year-old Japanese woman with a history of one miscarriage at 6 weeks' gestation had become pregnant under anticoagulation treatment of 2500 U danaparoid sodium because of her osteoporosis and osteonecrosis of femoral head and 100 mg aspirin per day from 4 weeks' gestation at Nagoya City University Hospital. She has had a 22-year history of SLE, which had been in remission for more than 4 years under a treatment of 5 mg prednisolone daily. 

At this point, our case was not diagnosed with APS, because she suffered from only one miscarriage. However, we planed to do anticoagulant treatment during pregnancy, because her lupus anticoagulant (LA) [8] was ascertained to be strongly positive by diluted activated partial thromboplastin time (aPTT) methods and diluted Russel's viper venom time (RVVT) before pregnancy. Prednisolone therapy was interrupted before this pregnancy because the patient thought it affected infertility.

Her edema and protein urea appeared at 17 weeks' gestation (Table 1). Her blood pressure was 121/78 mmHg and laboratory studies showed anti-*β*2-glycoprotein I (*β*2GPI) 9.4 U/mL (normal range < 1.9), aPTT 30%, LA by aPTT 30 seconds, LA by RVVT 1.47, and urinary protein 2.0 g/day at 17 weeks of gestation. At 21 weeks of gestation, she had body edema and protein urea (8.1 g/24 h). Laboratory studies were shown in Table 1. At 21 weeks of gestation she was hospitalized because of severe edema and acute renal dysfunction, and treatment of 40 mg prednisolone daily started at 22 weeks of gestation.

She had developed epigastralgia and vomiting. Thrombocytopenia and hemolytic anemia together with liver and renal dysfunction and an elevated level of C-reactive protein were observed (Table 1). At 22 weeks and 6 days of gestation, emergency cesarean section was performed because of fetal distress with loss of variability and severe late deceleration, abnormal Doppler flow velocimetry waveform analysis suggestive of fetal hypoxemia, and the HELLP syndrome. The baby was weighting 414 g, and the clinical manifestations were morphologically normal. Four days after birth the baby died of pulmonary hemorrhage. Placental infarction was pathologically found. 

Her laboratory examinations showed liver dysfunction, and the data from coagulation studies indicated the diagnostic criteria for DIC. Intravascular infusion of nafamostat methylate together with antibiotics, gamma-globulin 15 g, and platelet transfusion gradually improved the clinical symptoms and laboratory abnormalities. The patient had no fever or persistent epigastralgia. Her blood pressure was 140/92 mmHg and pulse was 118 per min under antihypertensive therapy. Her renal biopsy showed lupus nephritis INS/RPS class IV with mesangial interposition, massive subendothelial, subepithelial and mesangial deposit, fibrinoid necrosis, karyorrhexis, swelling of endothelial cells, and foam cells. The patient was diagnosed MAPS. Then multitarget therapy (prednisolone, cyclosporine, and mizoribine) was started.

Presently, the patient is now being treated as an outpatient who is asymptomatic, while continuing treatment with cyclosporine A (75 mg/day), mizoribine (100 mg/day), and prednisolone (25 mg/day). Anticoagulation with warfarin (2 mg/day) was started to a target international normalized ratio (INR) of 2-3. 

## 3. Discussion

MAPS have been reported as encompassing several conditions mainly affecting the microvasculature of selected organs: the liver in HELLP syndrome, kidney, brain and skin in TTP and DIC. The present case had HELLP syndrome, renal dysfunction, and DIC.

Several lines of evidence suggest that MAPS may be triggered, by mainly infections, trauma, surgery, anticoagulation withdrawal, malignancies, or lupus flare, or infrequently appear during pregnancy or puerperium [9, 10]. Pauzner et al. reported the relationship between liver infarction in HELLP syndrome and APS and indicated that hepatic infarction during pregnancy was almost always associated with APS [4]. Electronic microscope findings of her renal biopsy demonstrated evidence of microangiopathic, although we could not find liver infarction in the present case. 

The term thrombotic microangiopathic haemolytic anaemia (TMHA) was originally introduced in 1952 [11]. TMHA encompasses TTP and hemolytic-uremic syndrome (HUS). Espinosa et al. reviewed the association of aPL and TMHA comprehensively [12]. Simultaneous with the TMHA and aPL story came the association of patients with HELLP syndrome and aPL [13]. However, in the HELLP syndrome, hepatic infarctions are not uncommonly documented and are undoubtedly due to small vessel perturbation [13]. In our present case of MAPS with the HELLP syndrome, there were virtually no large vessel occlusions. 

The APS has the multifactorial pathogenesis though the criteria include arterial, venous, or small vessel thrombosis [1]. The first major player in MAPS might be the endothelial cells. Endothelial cell activation has been demonstrated in TTP, aPL and HELLP syndrome [14–17]. The pathogenicity of some aPL has been dramatically demonstrated in animal models [14]. The many complex pathways were involved in intracellular signaling resulting in the conversion of cells, particularly endothelial, to a prothrombotic state [15, 16]. The influence of these antibodies on monocytes [18], as well as on platelets antibodies is now also well described [19]. The aPL, are known to be heterogeneous in function and specificity and that more than one type may be present in any individual with APS. It is clear that APS is a multiorgan-multisystem disease with multiple possible clinical manifestations.

The aspirin and heparin combined therapy is effective in 70–80% of recurrent fetal loss patients with APS [20]. Our present case was treated with danaparoid instead of heparin to avoid osteoporosis because the patient experienced osteonecrosis of femoral head. Magnani HN reported an analysis of clinical outcomes of 91 pregnancies treated with danaparoid in 83 women with a history of thrombophilia and/or repeated pregnancy loss [21]. The successful birth rate and adverse event profile indicated that danaparoid can be an effective and safe alternative antithrombotic in pregnancies complicated by intolerance or resistance to low molecular-weight heparins. 

Among HELLP cases diagnosed antepartum, 90% are in the third trimester, and the syndrome rarely occurs before 27 weeks of gestation [6]. Early onset of HELLP syndrome and severe organ dysfunction are the characteristics of the treatment failure of obstetrical APS. Target therapy not for thrombosis but for microangiopathy is needed for patients with APS against anticoagulant therapy. 

## Figures and Tables

**Table 1:** Laboratory data (abnormal findings are bold).

	17W5D	21W1D	22W6D C/S	Postpartum (Day 14)	Normal range
Blood count					
WBC (*μ*L)	5200	6100	5600	5000	3000–8500
RBC (*μ*L)	**2.87**	**2.65**	**2.51**	**2.07**	3.78–4.99 × 10^6^
Hb (g/dL)	**9.2**	**8.6**	**7.9**	**6.5**	10.8–14.9
Ht (%)	**27.3**	**25.6**	**23.4**	**19.9**	35.6–45.4
Plt (*μ*L)	168	**144**	**55**	**133**	150–361 × 10^3^
Coagulation test					
aPTT (%)		**48.6**	**49.6**	94.2	76–130
PT (%)		**136.5**	**134.2**	**134.2**	70–130
Fibrinogen (mg/dL)		**441**	**414**	206	200–400
FDP (*μ*g/mL)		**12.6**	**38.5**	**29.9**	<5.0
D-dimer (*μ*g/mL)		**6.6**	**24.5**	**22.1**	<1.0
Immunological data					
CRP (mg/dL)	<0.3	**0.54**	**1.78**	<0.3	<0.30
C3 (mg/dL)	55	**36**	**39**	**35**	68–128
C4 (mg/dL)	14	16	17	17	13–36
CH50 (U/mL)	37.2	**28.4**	**29.3**	33.5	32.0–48.0
Anti-dsDNA Ab (IU/mL)	**50**	**38**	**43**	**28**	<12
Anti-ssDNA Ab (AU/mL)		**560**	**800**	**412**	<25
Anti-Sm Ab (index)	<5.0	<5.0	<5.0	<5.0	<7.0
ANA (index)	**1280**	**640**	**640**		<40
Serum chemistry					
TP (g/dL)	**5.5**	**4.4**	**3.9**	**3.7**	6.7–8.3
AST (IU/L)	20	27	**165**	20	13–33
ALT (IU/L)	11	8	**70**	9	6–27
LDH (IU/L)	**260**	**268**	**495**	**343**	119–229
ALP (IU/L)	210	232	297	200	115–359
T-Bil (mg/dL)	0.4	0.4			0.3–1.2
BUN (mg/dL)	18	**41**	**56**	**30**	8–22
Creatinine (mg/dL)	**0.8**	**1.9**	**3.3**	**2.3**	0.4–0.7
Uric acid (mg/dL)	**7.3**	**11.3**	**11.3**	**9.6 **	2.3–7.0
Blood pressure (mmHg)	121/78	**150/80**	**160/95**	**154/84**	
Urine					
Urinary protein (g/day)	**2**	**8.1**	**4.4**	**9.1**	0.03–0.12
Urinary sugar	Negative	Negative	Negative	Negative	
